# Personal

**Published:** 1901-07

**Authors:** 


### PERSONAL.

Dr J. D. Griffith, of Kansas City, was elected president of the Medical Association of Missouri at the annual meeting held at Jefferson City, May 21-23, 1901. This election caused universal satisfaction as Dr. Griffith is one of the most capable men in his profession in the State of Missouri, and undei his administration a successful medical year is predicted.

Dr. Frank L. Christian, formerly a house physician at Bellevue Hospital, New York, and more lately medical officer of the Eastern District Reformatory, has been appointed medical superintendent of the Elmira Reformatory. The position carries a salary of $3,500 a year and a residence.

Dr. Frederick H. Millener, of Buffalo, has removed from No. 5 North Pearl Street to No. 724 Main Street. Practice limited to diseases of the ear, nose and throat. Telephone, Tupper 777.

Dr. W. F. Beck, U. of B., '93, of Vermillion, O., has removed to Buffalo. His office and residence are at 80 Lemon Street.

Dr. Henry L. Elsner, of Syracuse, President of the Medical Society of the State of New York, attended the semiannual meeting of the Medical Society of the County of Erie, June 11, 1901, at which time he read a paper entitled, Malignant endocarditis. Dr. William C. Krauss entertained a group of physicians in Dr. Elsner's honor at luncheon at the Buffalo Club after the meeting adjourned.

Dr. A. Walter Suiter, of Herkimer, a former president of the Medical Society of the State of New York, attended the semiannual meeting of the Medical Society of the County of Erie, June 11, 1901. He spent a few days in Buffalo on his way home from St. Paul in the interests of the American Public Health Association, which will hold its annual meeting in Buffalo next September.

Mr. Thomas Stoddart, of Buffalo, was elected president of the New York State Pharmaceutical Association at its annual meeting held in Buffalo, June 4-7, 1901. Commenting on the choice of Mr. Stoddart the Buffalo *Evening News* of June 7, most fittingly said:

Buffalo is much gratified that the State Pharmaceutical Association should have chosen its honored citizen, Mr. Thomas Stoddart, to be its presiding officer for the ensuing year. Because of the high standard of the individual membership of the State Association, the selection comes as an honor to Mr. Stoddart and yet the association, at the same time, honors itself in the choice. Both as a business man and as a citizen Mr. Stoddart is a representative of whom Buffalo feels justly proud and the pharmacists and the new president are alike to be congratulated on the outcome of the balloting.

Dr. Herbert M. Tolfree, of Buffalo, has been appointed to the medical corps of the navy and will be commissioned as assistant surgeon. Dr. Tolfree was a naval cadet and took up the study of medicine to enter the medical corps.

Dr. Edwin Ricketts, of Cincinnati, has removed his office from 415 to 408 Broadway. Hours: 10 to 11 a.m., 1 to 3 p.m., and by appointment. Office telephone, Main 118; residence, N. 391 R.

Dr. L. G. Waterman, of Centerville, N.Y., has removed to Buffalo. His office and residence are at 54 Indian Church Road. Hours: 8 to 10, and 6 to 8.

